# A brand-new era has begun for JECT

**DOI:** 10.1051/ject/2023006

**Published:** 2023-03-24

**Authors:** Raymond K Wong

Last November, we announced some important changes for the Journal of Extra-Corporeal Technology. The moment has arrived! As of this issue, JECT is now Open Access! All new articles are now immediately available to all readers as soon as they are published on our brand-new website (https://ject.edpsciences.org). Individual articles are available in full HTML, PDF, and ePUB format, adapted for consultation on different devices. Full issues are also available in a “flipbook” format. In fact the PDFs of accepted articles are also available ahead-of-press, in a pre-publication state.

JECT has a refreshed logo and a brand-new cover, giving the journal a modern look and feel.

Old vs. new logo


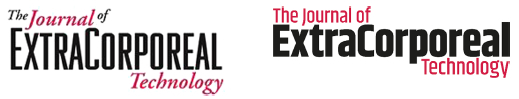


Old vs. new cover


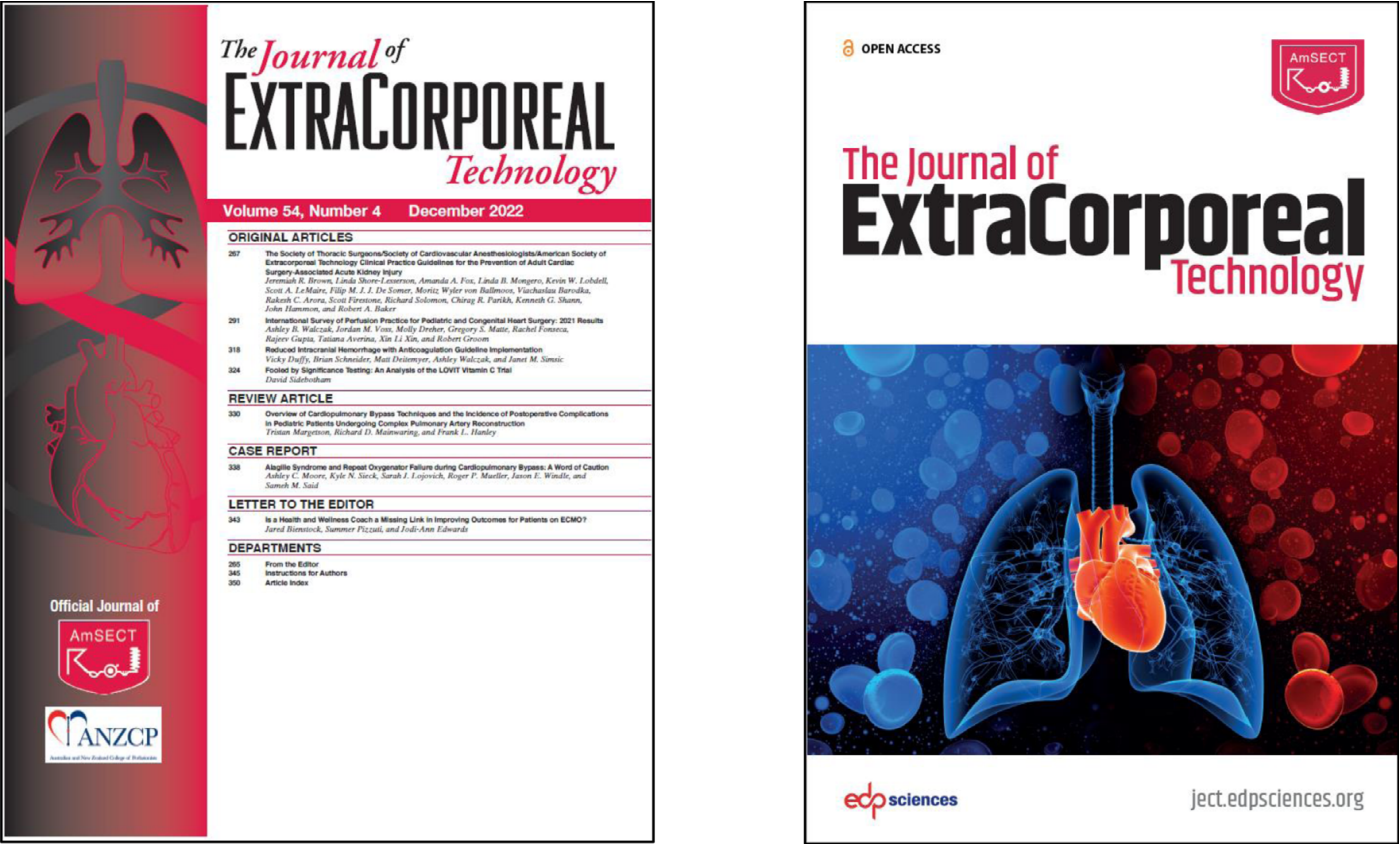


Along with the flashy features, we also have not so flashy brand-new policies for our authors in our revamped instructions. Things like 1) copyright (which now belongs to authors, CC BY 4.0); 2) new processes to support efficient pre-print publication online after manuscript acceptance; 3) guidance to authors to use linked public data repositories for supplemental data and 4) new disclosure declaration requirements at the end of the article to comply with best practices and meet demands by indexation databases (see “Instructions for Authors” for all details).

There are many people to thank for shepherding this huge transition for the journal. Firstly, I thank the AmSECT board of directors and the journal leadership team for initially greenlighting the efforts to modernize our journal and to ultimately approve our new publication partnership. Secondly, I thank AmSECT’s Management Company, Smithbucklin and Riverwinds Consulting whose members were critical in guiding us through the considerations of our new needs and the vetting process. Last but not least, I am most grateful to the team at our new publication partners, EDP Sciences who have diligently ensured that all the needs for the transition are being addressed, both the flashy (with creative help from their web design and marketing teams) and the mundane (policies). Even though our new partners are based a continent away in France, I am happy to report that communication has not been an issue and we have been collaborating efficiently and smoothly!

In this issue, Lohbusch, et al. [1] reports on a recent survey of adult clinical perfusion practices in the United States. Although geographically limited to the Eastern United States, it nicely captures a snapshot of the techniques and devices currently being utilized. The survey also identifies opportunities for improving the adoption of best practice guidelines published by various groups recently. On the other side of the country, Sleasman, et al. [2] describe a pilot program to test a novel online ECMO supply sharing platform hosted by the Extracorporeal Life Support Organization (ELSO). Nine regional centers successfully participated in an effort to alleviate supply shortages amongst themselves. Propagated internationally by ELSO, this platform has the potential to meaningfully impact future pandemics, at least from the perspective of equipment and supply availability.

Our first issue in this new era is not too lengthy as we transition, but we look forward to advancing our journal on all fronts as we execute on positioning all the proper pieces in place.


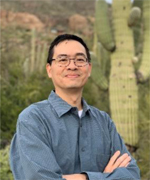


Raymond K. Wong at The University of Arizona.

https://www.arizona.edu/about#land-acknowledgment.
